# (*Z*)-3-(4-Bromo­phen­yl)-2-[(2-phenyl­cyclo­hex-2-en-1-yl)imino]-1,3-thia­zol­idin-4-one

**DOI:** 10.1107/S1600536812024646

**Published:** 2012-06-02

**Authors:** Chin Wei Ooi, Hoong-Kun Fun, Ching Kheng Quah, Murugan Sathishkumar, Alagusundaram Ponnuswamy

**Affiliations:** a X-ray Crystallography Unit, School of Physics, Universiti Sains Malaysia, 11800 USM, Penang, Malaysia; b Department of Organic Chemistry, School of Chemistry, Madurai Kamaraj University, Madurai 625 021, Tamil Nadu, India

## Abstract

The title compound, C_21_H_19_BrN_2_OS, exists in a *cis* conformation with respect to the N=C bond [1.2602 (14) Å]. The cyclo­hexene ring adopts a distorted half-chair conformation and the C—N bond lies in an equatorial orientation. The thia­zolidine ring forms dihedral angles of 53.76 (7) and 57.22 (7)° with the benzene and bromo-substituted benzene rings, respectively. The dihedral angle between the benzene and bromo-substituted benzene rings is 76.06 (7)°. In the crystal, inversion dimers linked by pairs of C—H⋯O hydrogen bonds generate *R*_2_^2^(14) loops. The crystal is further consolidated by weak C—H⋯π inter­actions.

## Related literature

For related structures and background to thia­zolidin-4-one derivatives, see: Fun *et al.* (2011 ▶); Ooi *et al.* (2012*a* ▶,*b* ▶). For hydrogen-bond motifs, see: Bernstein *et al.* (1995 ▶). For ring conformations, see: Cremer & Pople (1975 ▶). For the stability of the temperature controller used in the data collection, see: Cosier & Glazer (1986 ▶).
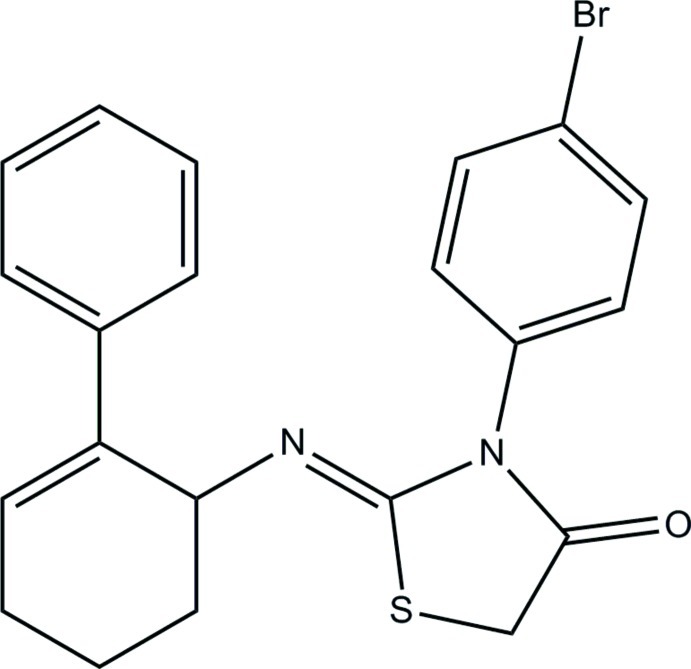


## Experimental

### 

#### Crystal data

C_21_H_19_BrN_2_OS
*M* *_r_* = 427.35
Monoclinic, 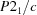

*a* = 9.4573 (1) Å
*b* = 16.6662 (3) Å
*c* = 13.8812 (2) Å
β = 122.665 (1)°
*V* = 1841.88 (5) Å^3^
*Z* = 4
Mo *K*α radiation
μ = 2.36 mm^−1^
*T* = 100 K
0.45 × 0.29 × 0.25 mm

#### Data collection

Bruker SMART APEXII CCD diffractometer
Absorption correction: multi-scan (*SADABS*; Bruker, 2009 ▶) *T*_min_ = 0.418, *T*_max_ = 0.587
24632 measured reflections
6727 independent reflections
5751 reflections with *I* > 2σ(*I*)
*R*_int_ = 0.021

#### Refinement

*R*[*F* ^2^ > 2σ(*F* ^2^)] = 0.027
*wR*(*F* ^2^) = 0.069
*S* = 1.04
6727 reflections
235 parameters
H-atom parameters constrained
Δρ_max_ = 0.55 e Å^−3^
Δρ_min_ = −0.27 e Å^−3^

### 

Data collection: *APEX2* (Bruker, 2009 ▶); cell refinement: *SAINT* (Bruker, 2009 ▶); data reduction: *SAINT*; program(s) used to solve structure: *SHELXTL* (Sheldrick, 2008 ▶); program(s) used to refine structure: *SHELXTL*; molecular graphics: *SHELXTL*; software used to prepare material for publication: *SHELXTL* and *PLATON* (Spek, 2009 ▶).

## Supplementary Material

Crystal structure: contains datablock(s) global, I. DOI: 10.1107/S1600536812024646/hb6826sup1.cif

Structure factors: contains datablock(s) I. DOI: 10.1107/S1600536812024646/hb6826Isup2.hkl

Supplementary material file. DOI: 10.1107/S1600536812024646/hb6826Isup3.cml

Additional supplementary materials:  crystallographic information; 3D view; checkCIF report

## Figures and Tables

**Table 1:** Hydrogen-bond geometry (Å, °) *Cg*1 is the centroid of the C1–C6 benzene ring.

*D*—H⋯*A*	*D*—H	H⋯*A*	*D*⋯*A*	*D*—H⋯*A*
C18—H18*A*⋯O1^i^	0.93	2.33	3.2333 (15)	164
C17—H17*A*⋯*Cg*1^ii^	0.93	2.88	3.5802 (15)	133

Symmetry codes: (i) 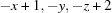; (ii) 
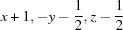
.

**Fig. 1.**
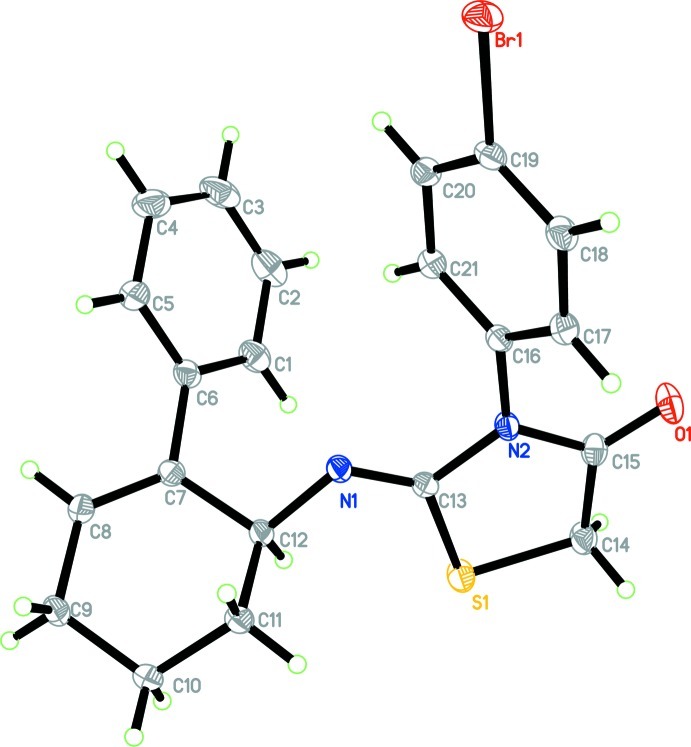
The molecular structure of the title compound, showing 50% probability displacement ellipsoids.

**Fig. 2.**
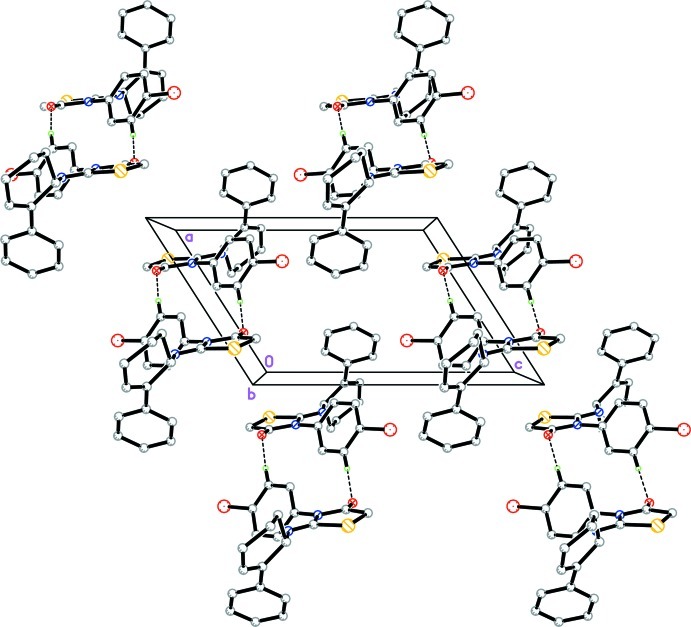
The crystal packing of the title compound, viewed along the b axis. H atoms not involved in the intermolecular interactions (dashed lines) have been omitted for clarity.

## supplementary crystallographic information

### Comment

As part of our ongoing studies of thiazolidin-4-one derivatives (Fun *et al.*, 2011; Ooi *et al.*, 2012*a*,*b*), we now describe the structure of the title compound.

The title compound (Fig. 1) exists in *cis* configuration with respect to the N1 ═C13 bond [N1 ═C13 = 1.2602 (14) Å]. The cyclohexene (C7–C12) ring adopts a distorted sofa conformation and the puckering parameters are Q = 0.4857 (14) Å, θ = 131.97 (17)° and φ = 42.1 (2)° (Cremer & Pople, 1975). The thiazolidine (S1/N2/C13–C15) ring is essentially planar with a maximum deviation of 0.019 (2) Å at atom C15 and forms dihedral angles of 53.76 (7) and 57.22 (7)° respectively with the benzene ring (C1–C6) and bromo-substituted benzene ring (C16–C21). The dihedral angle between the benzene ring and bromo-substituted benzene ring is 76.06 (7)°. The bond lengths and angles are comparable to related structures (Fun *et al.*, 2011; Ooi *et al.*, 2012*a*&*b*).

In the crystal (Fig. 2), pairs of C18—H18A···O1 hydrogen bonds (Table 1) link the neighbouring molecules to form dimers, generating *R*_2_^2^ (14) ring motifs (Bernstein *et al.*, 1995). The crystal is further consolidated by C17—H17A···*Cg*1 interactions (Table 1), involving the centroid of the benzene ring (C1–C6; *Cg*1).

### Experimental

A mixture of 1-(4-bromophenyl)-3-(2-phenylcyclohex-2-enyl)thiourea (0.5 g, 2.3 mmol) and chloroacetyl chloride (0.29 g, 4.6 mmol) was heated to reflux in 1,4-dioxane (10 ml) at 100°C for 5 h. The reaction mixture was washed with diluted sodium bicarbonate solution (25 ml) and dried over anhydrous sodium sulfate. The solvent was then evaporated under reduced pressure and the resulting residue was subjected to column chromatography using silica gel (60–120 mesh) as the stationary phase and petroleum ether-ethyl acetate (90:10) as the mobile phase to give the pure product. Yield: 0.74 g (75%); *M.p.*: 172–173°C. Yellow blocks were obtained by recrystallization from dichloromethane solution.

### Refinement

All the H atoms were positioned geometrically and refined using a riding model with *U*_iso_(H) = 1.2 *U*_eq_(C) (C—H = 0.93, 0.97 and 0.98 Å).

### Crystal data

**Table:**

C_21_H_19_BrN_2_OS	*F*(000) = 872
*M**_r_* = 427.35	*D*_x_ = 1.541 Mg m^−^^3^
Monoclinic, *P*2_1_/*c*	Mo *K*α radiation, λ = 0.71073 Å
Hall symbol: -P 2ybc	Cell parameters from 9954 reflections
*a* = 9.4573 (1) Å	θ = 2.8–32.6°
*b* = 16.6662 (3) Å	µ = 2.36 mm^−^^1^
*c* = 13.8812 (2) Å	*T* = 100 K
β = 122.665 (1)°	Block, yellow
*V* = 1841.88 (5) Å^3^	0.45 × 0.29 × 0.25 mm
*Z* = 4	

### Data collection

**Table:**

Bruker SMART APEXII CCD diffractometer	6727 independent reflections
Radiation source: fine-focus sealed tube	5751 reflections with *I* > 2σ(*I*)
Graphite monochromator	*R*_int_ = 0.021
φ and ω scans	θ_max_ = 32.7°, θ_min_ = 2.1°
Absorption correction: multi-scan (*SADABS*; Bruker, 2009)	*h* = −14→12
*T*_min_ = 0.418, *T*_max_ = 0.587	*k* = −25→25
24632 measured reflections	*l* = −21→20

### Refinement

**Table:**

Refinement on *F*^2^	Primary atom site location: structure-invariant direct methods
Least-squares matrix: full	Secondary atom site location: difference Fourier map
*R*[*F*^2^ > 2σ(*F*^2^)] = 0.027	Hydrogen site location: inferred from neighbouring sites
*wR*(*F*^2^) = 0.069	H-atom parameters constrained
*S* = 1.04	*w* = 1/[σ^2^(*F*_o_^2^) + (0.0366*P*)^2^ + 0.4872*P*] where *P* = (*F*_o_^2^ + 2*F*_c_^2^)/3
6727 reflections	(Δ/σ)_max_ = 0.002
235 parameters	Δρ_max_ = 0.55 e Å^−^^3^
0 restraints	Δρ_min_ = −0.27 e Å^−^^3^

### Special details

**Table:**

Experimental. The crystal was placed in the cold stream of an Oxford Cryosystems Cobra open-flow nitrogen cryostat (Cosier & Glazer, 1986) operating at 100 (1) K.
Geometry. All e.s.d.'s (except the e.s.d. in the dihedral angle between two l.s. planes) are estimated using the full covariance matrix. The cell e.s.d.'s are taken into account individually in the estimation of e.s.d.'s in distances, angles and torsion angles; correlations between e.s.d.'s in cell parameters are only used when they are defined by crystal symmetry. An approximate (isotropic) treatment of cell e.s.d.'s is used for estimating e.s.d.'s involving l.s. planes.
Refinement. Refinement of *F*^2^ against ALL reflections. The weighted *R*-factor *wR* and goodness of fit *S* are based on *F*^2^, conventional *R*-factors *R* are based on *F*, with *F* set to zero for negative *F*^2^. The threshold expression of *F*^2^ > σ(*F*^2^) is used only for calculating *R*-factors(gt) *etc*. and is not relevant to the choice of reflections for refinement. *R*-factors based on *F*^2^ are statistically about twice as large as those based on *F*, and *R*- factors based on ALL data will be even larger.

### Fractional atomic coordinates and isotropic or equivalent isotropic displacement parameters (Å^2^)

**Table:**

	*x*	*y*	*z*	*U*_iso_*/*U*_eq_	
Br1	0.262828 (17)	−0.045374 (7)	0.626813 (11)	0.02221 (4)	
S1	0.23445 (4)	0.328421 (18)	1.04486 (3)	0.02081 (7)	
O1	0.31396 (12)	0.10045 (6)	1.08962 (8)	0.02302 (18)	
N1	0.19965 (12)	0.30829 (6)	0.83807 (8)	0.01380 (17)	
N2	0.26458 (12)	0.19597 (6)	0.95606 (8)	0.01426 (17)	
C1	−0.18227 (16)	0.31078 (8)	0.68851 (12)	0.0211 (2)	
H1A	−0.1535	0.3282	0.7605	0.025*	
C2	−0.30028 (16)	0.25012 (8)	0.63418 (13)	0.0260 (3)	
H2A	−0.3507	0.2276	0.6697	0.031*	
C3	−0.34355 (17)	0.22278 (8)	0.52691 (13)	0.0305 (3)	
H3A	−0.4219	0.1818	0.4910	0.037*	
C4	−0.26912 (18)	0.25702 (9)	0.47385 (12)	0.0295 (3)	
H4A	−0.2979	0.2392	0.4020	0.035*	
C5	−0.15163 (16)	0.31794 (8)	0.52797 (10)	0.0217 (2)	
H5A	−0.1024	0.3405	0.4917	0.026*	
C6	−0.10613 (14)	0.34592 (7)	0.63606 (10)	0.0164 (2)	
C7	0.02578 (14)	0.40843 (7)	0.69463 (9)	0.01424 (19)	
C8	0.02723 (15)	0.47371 (7)	0.63914 (10)	0.0167 (2)	
H8A	−0.0613	0.4805	0.5636	0.020*	
C9	0.16181 (16)	0.53696 (7)	0.69010 (10)	0.0173 (2)	
H9A	0.1092	0.5895	0.6721	0.021*	
H9B	0.2270	0.5332	0.6552	0.021*	
C10	0.27966 (16)	0.52939 (7)	0.81938 (10)	0.0180 (2)	
H10A	0.2274	0.5531	0.8565	0.022*	
H10B	0.3828	0.5584	0.8443	0.022*	
C11	0.32010 (15)	0.44150 (7)	0.85432 (10)	0.0173 (2)	
H11A	0.3981	0.4380	0.9365	0.021*	
H11B	0.3736	0.4180	0.8179	0.021*	
C12	0.16053 (14)	0.39451 (7)	0.81969 (9)	0.01417 (19)	
H12A	0.1183	0.4119	0.8671	0.017*	
C13	0.23002 (14)	0.27822 (7)	0.93069 (9)	0.01370 (19)	
C14	0.28566 (16)	0.23800 (8)	1.12881 (10)	0.0194 (2)	
H14A	0.3938	0.2437	1.2000	0.023*	
H14B	0.2014	0.2276	1.1468	0.023*	
C15	0.29094 (15)	0.16970 (7)	1.05911 (10)	0.0163 (2)	
C16	0.26532 (14)	0.14088 (7)	0.87679 (9)	0.01387 (19)	
C17	0.40768 (15)	0.09488 (7)	0.91258 (10)	0.0172 (2)	
H17A	0.5026	0.1016	0.9857	0.021*	
C18	0.40766 (16)	0.03854 (7)	0.83822 (11)	0.0182 (2)	
H18A	0.5018	0.0070	0.8610	0.022*	
C19	0.26453 (15)	0.03056 (7)	0.72975 (10)	0.0161 (2)	
C20	0.12226 (15)	0.07709 (7)	0.69269 (10)	0.0165 (2)	
H20A	0.0279	0.0708	0.6192	0.020*	
C21	0.12320 (14)	0.13315 (7)	0.76719 (10)	0.0154 (2)	
H21A	0.0296	0.1652	0.7439	0.018*	

### Atomic displacement parameters (Å^2^)

**Table:**

	*U*^11^	*U*^22^	*U*^33^	*U*^12^	*U*^13^	*U*^23^
Br1	0.02917 (7)	0.01757 (6)	0.02310 (7)	0.00051 (4)	0.01621 (6)	−0.00474 (4)
S1	0.03491 (17)	0.01512 (13)	0.01733 (13)	0.00423 (11)	0.01734 (13)	0.00082 (10)
O1	0.0320 (5)	0.0179 (4)	0.0258 (4)	0.0073 (4)	0.0200 (4)	0.0086 (3)
N1	0.0161 (4)	0.0116 (4)	0.0139 (4)	0.0011 (3)	0.0082 (3)	0.0007 (3)
N2	0.0187 (4)	0.0120 (4)	0.0137 (4)	0.0016 (3)	0.0098 (4)	0.0018 (3)
C1	0.0189 (5)	0.0175 (5)	0.0273 (6)	0.0013 (4)	0.0127 (5)	0.0019 (4)
C2	0.0164 (5)	0.0196 (6)	0.0399 (8)	0.0008 (4)	0.0139 (5)	0.0046 (5)
C3	0.0171 (6)	0.0174 (6)	0.0401 (8)	−0.0024 (5)	0.0042 (6)	−0.0016 (5)
C4	0.0245 (6)	0.0219 (6)	0.0236 (6)	0.0002 (5)	0.0007 (5)	−0.0047 (5)
C5	0.0227 (6)	0.0183 (5)	0.0179 (5)	−0.0001 (4)	0.0068 (5)	0.0002 (4)
C6	0.0141 (5)	0.0129 (5)	0.0183 (5)	0.0021 (4)	0.0062 (4)	0.0015 (4)
C7	0.0148 (5)	0.0133 (5)	0.0146 (5)	0.0008 (4)	0.0079 (4)	−0.0002 (4)
C8	0.0182 (5)	0.0156 (5)	0.0141 (5)	0.0011 (4)	0.0074 (4)	0.0013 (4)
C9	0.0217 (5)	0.0131 (5)	0.0181 (5)	0.0002 (4)	0.0113 (4)	0.0018 (4)
C10	0.0205 (5)	0.0127 (5)	0.0180 (5)	−0.0023 (4)	0.0086 (4)	−0.0012 (4)
C11	0.0170 (5)	0.0137 (5)	0.0168 (5)	−0.0002 (4)	0.0062 (4)	0.0003 (4)
C12	0.0173 (5)	0.0112 (5)	0.0138 (4)	0.0011 (4)	0.0083 (4)	0.0009 (3)
C13	0.0150 (5)	0.0118 (5)	0.0143 (4)	0.0005 (4)	0.0079 (4)	−0.0005 (3)
C14	0.0268 (6)	0.0180 (5)	0.0164 (5)	0.0025 (4)	0.0137 (5)	0.0021 (4)
C15	0.0172 (5)	0.0183 (5)	0.0157 (5)	0.0028 (4)	0.0104 (4)	0.0034 (4)
C16	0.0172 (5)	0.0113 (5)	0.0149 (5)	0.0003 (4)	0.0099 (4)	0.0004 (3)
C17	0.0176 (5)	0.0153 (5)	0.0171 (5)	0.0022 (4)	0.0082 (4)	0.0014 (4)
C18	0.0201 (5)	0.0154 (5)	0.0204 (5)	0.0041 (4)	0.0118 (5)	0.0017 (4)
C19	0.0217 (5)	0.0115 (5)	0.0189 (5)	−0.0010 (4)	0.0134 (5)	−0.0014 (4)
C20	0.0183 (5)	0.0152 (5)	0.0167 (5)	−0.0013 (4)	0.0099 (4)	−0.0004 (4)
C21	0.0158 (5)	0.0144 (5)	0.0166 (5)	0.0006 (4)	0.0092 (4)	0.0007 (4)

### Geometric parameters (Å, º)

**Table:**

Br1—C19	1.9023 (11)	C8—H8A	0.9300
S1—C13	1.7725 (11)	C9—C10	1.5230 (17)
S1—C14	1.8039 (12)	C9—H9A	0.9700
O1—C15	1.2080 (14)	C9—H9B	0.9700
N1—C13	1.2602 (14)	C10—C11	1.5254 (16)
N1—C12	1.4714 (14)	C10—H10A	0.9700
N2—C15	1.3839 (14)	C10—H10B	0.9700
N2—C13	1.4090 (14)	C11—C12	1.5293 (16)
N2—C16	1.4360 (14)	C11—H11A	0.9700
C1—C2	1.3884 (18)	C11—H11B	0.9700
C1—C6	1.3992 (17)	C12—H12A	0.9800
C1—H1A	0.9300	C14—C15	1.5122 (17)
C2—C3	1.391 (2)	C14—H14A	0.9700
C2—H2A	0.9300	C14—H14B	0.9700
C3—C4	1.387 (2)	C16—C17	1.3875 (16)
C3—H3A	0.9300	C16—C21	1.3894 (15)
C4—C5	1.3887 (19)	C17—C18	1.3953 (16)
C4—H4A	0.9300	C17—H17A	0.9300
C5—C6	1.3990 (17)	C18—C19	1.3846 (17)
C5—H5A	0.9300	C18—H18A	0.9300
C6—C7	1.4858 (16)	C19—C20	1.3889 (17)
C7—C8	1.3374 (16)	C20—C21	1.3902 (16)
C7—C12	1.5177 (15)	C20—H20A	0.9300
C8—C9	1.5032 (17)	C21—H21A	0.9300
			
C13—S1—C14	92.97 (5)	C10—C11—H11A	109.5
C13—N1—C12	117.57 (9)	C12—C11—H11A	109.5
C15—N2—C13	117.01 (9)	C10—C11—H11B	109.5
C15—N2—C16	121.13 (9)	C12—C11—H11B	109.5
C13—N2—C16	121.80 (9)	H11A—C11—H11B	108.1
C2—C1—C6	120.71 (13)	N1—C12—C7	108.97 (9)
C2—C1—H1A	119.6	N1—C12—C11	109.42 (9)
C6—C1—H1A	119.6	C7—C12—C11	110.99 (9)
C1—C2—C3	120.39 (13)	N1—C12—H12A	109.1
C1—C2—H2A	119.8	C7—C12—H12A	109.1
C3—C2—H2A	119.8	C11—C12—H12A	109.1
C4—C3—C2	119.56 (12)	N1—C13—N2	122.43 (10)
C4—C3—H3A	120.2	N1—C13—S1	127.37 (9)
C2—C3—H3A	120.2	N2—C13—S1	110.21 (8)
C3—C4—C5	120.05 (13)	C15—C14—S1	107.80 (8)
C3—C4—H4A	120.0	C15—C14—H14A	110.1
C5—C4—H4A	120.0	S1—C14—H14A	110.1
C4—C5—C6	121.14 (13)	C15—C14—H14B	110.1
C4—C5—H5A	119.4	S1—C14—H14B	110.1
C6—C5—H5A	119.4	H14A—C14—H14B	108.5
C5—C6—C1	118.15 (11)	O1—C15—N2	124.24 (11)
C5—C6—C7	120.14 (11)	O1—C15—C14	123.83 (10)
C1—C6—C7	121.65 (11)	N2—C15—C14	111.93 (10)
C8—C7—C6	121.45 (10)	C17—C16—C21	121.27 (10)
C8—C7—C12	121.33 (10)	C17—C16—N2	118.95 (10)
C6—C7—C12	117.22 (9)	C21—C16—N2	119.76 (10)
C7—C8—C9	124.66 (10)	C16—C17—C18	119.62 (11)
C7—C8—H8A	117.7	C16—C17—H17A	120.2
C9—C8—H8A	117.7	C18—C17—H17A	120.2
C8—C9—C10	113.05 (9)	C19—C18—C17	118.61 (11)
C8—C9—H9A	109.0	C19—C18—H18A	120.7
C10—C9—H9A	109.0	C17—C18—H18A	120.7
C8—C9—H9B	109.0	C18—C19—C20	122.15 (11)
C10—C9—H9B	109.0	C18—C19—Br1	119.19 (9)
H9A—C9—H9B	107.8	C20—C19—Br1	118.65 (9)
C9—C10—C11	110.65 (9)	C19—C20—C21	118.93 (11)
C9—C10—H10A	109.5	C19—C20—H20A	120.5
C11—C10—H10A	109.5	C21—C20—H20A	120.5
C9—C10—H10B	109.5	C16—C21—C20	119.40 (11)
C11—C10—H10B	109.5	C16—C21—H21A	120.3
H10A—C10—H10B	108.1	C20—C21—H21A	120.3
C10—C11—C12	110.87 (10)		
			
C6—C1—C2—C3	0.61 (19)	C15—N2—C13—N1	−178.09 (11)
C1—C2—C3—C4	−0.6 (2)	C16—N2—C13—N1	−0.77 (17)
C2—C3—C4—C5	0.3 (2)	C15—N2—C13—S1	1.31 (12)
C3—C4—C5—C6	0.0 (2)	C16—N2—C13—S1	178.64 (8)
C4—C5—C6—C1	0.04 (18)	C14—S1—C13—N1	179.95 (11)
C4—C5—C6—C7	177.08 (12)	C14—S1—C13—N2	0.58 (9)
C2—C1—C6—C5	−0.33 (18)	C13—S1—C14—C15	−2.07 (9)
C2—C1—C6—C7	−177.32 (11)	C13—N2—C15—O1	176.41 (11)
C5—C6—C7—C8	45.66 (16)	C16—N2—C15—O1	−0.94 (18)
C1—C6—C7—C8	−137.41 (12)	C13—N2—C15—C14	−2.97 (14)
C5—C6—C7—C12	−133.41 (11)	C16—N2—C15—C14	179.69 (10)
C1—C6—C7—C12	43.52 (15)	S1—C14—C15—O1	−176.23 (10)
C6—C7—C8—C9	−176.02 (11)	S1—C14—C15—N2	3.15 (12)
C12—C7—C8—C9	3.01 (18)	C15—N2—C16—C17	−58.28 (15)
C7—C8—C9—C10	−11.99 (17)	C13—N2—C16—C17	124.50 (12)
C8—C9—C10—C11	40.11 (14)	C15—N2—C16—C21	120.22 (12)
C9—C10—C11—C12	−60.99 (13)	C13—N2—C16—C21	−57.00 (15)
C13—N1—C12—C7	−142.56 (10)	C21—C16—C17—C18	−1.30 (17)
C13—N1—C12—C11	95.92 (12)	N2—C16—C17—C18	177.17 (10)
C8—C7—C12—N1	−143.05 (11)	C16—C17—C18—C19	0.38 (17)
C6—C7—C12—N1	36.02 (13)	C17—C18—C19—C20	0.48 (18)
C8—C7—C12—C11	−22.49 (15)	C17—C18—C19—Br1	179.52 (9)
C6—C7—C12—C11	156.58 (10)	C18—C19—C20—C21	−0.43 (18)
C10—C11—C12—N1	171.22 (9)	Br1—C19—C20—C21	−179.48 (9)
C10—C11—C12—C7	50.93 (13)	C17—C16—C21—C20	1.35 (17)
C12—N1—C13—N2	178.28 (10)	N2—C16—C21—C20	−177.11 (10)
C12—N1—C13—S1	−1.01 (15)	C19—C20—C21—C16	−0.48 (17)

### Hydrogen-bond geometry (Å, º)

Cg1 is the centroid of the C1–C6 benzene ring.

**Table:**

*D*—H···*A*	*D*—H	H···*A*	*D*···*A*	*D*—H···*A*
C18—H18*A*···O1^i^	0.93	2.33	3.2333 (15)	164
C17—H17*A*···*Cg*1^ii^	0.93	2.88	3.5802 (15)	133

Symmetry codes: (i) −*x*+1, −*y*, −*z*+2; (ii) *x*+1, −*y*−1/2, *z*−1/2.
